# Supplementary material for: Modeling glioblastoma heterogeneity as a dynamic network of cell states
Source: Mol Syst Biol. 2021 Sep 16;17(9):e10105. doi: 10.15252/msb.202010105 (PMC8444284; doi:10.15252/msb.202010105)
Supplement: Supplementary file 6 — Source Data for Figure 5 [file MSB-17-e10105-s004.zip › Figure5A_sourcedata/GSEA_3017/nosigdb_stateC.GseaPreranked.1622795002785/RICHARDS_NOWAKOWSKI_SCIENCE_2017_IPC_DIV1_UPREG.html]

Details for gene set RICHARDS\_NOWAKOWSKI\_SCIENCE\_2017\_IPC\_DIV1\_UPREG[GSEA]

|  || Dataset | state13017 |
| Phenotype | NoPhenotypeAvailable |
| Upregulated in class | na\_pos |
| GeneSet | RICHARDS\_NOWAKOWSKI\_SCIENCE\_2017\_IPC\_DIV1\_UPREG |
| Enrichment Score (ES) | 0.6466721 |
| Normalized Enrichment Score (NES) | 5.340267 |
| Nominal p-value | 0.0 |
| FDR q-value | 0.0 |
| FWER p-Value | 0.0 |
Table: GSEA Results Summary

  

Fig 1: Enrichment plot: RICHARDS\_NOWAKOWSKI\_SCIENCE\_2017\_IPC\_DIV1\_UPREG      
 Profile of the Running ES Score & Positions of GeneSet Members on the Rank Ordered List

  

| PROBE | GENE SYMBOL | GENE\_TITLE | RANK IN GENE LIST | RANK METRIC SCORE | RUNNING ES | CORE ENRICHMENT || 1 | SYNE2 |  |  | 0 | 0.839 | 0.0214 | Yes |
| 2 | TFDP2 |  |  | 1 | 0.787 | 0.0415 | Yes |
| 3 | HES6 |  |  | 5 | 0.694 | 0.0531 | Yes |
| 4 | MAGI1 |  |  | 6 | 0.652 | 0.0697 | Yes |
| 5 | ATAD2 |  |  | 7 | 0.649 | 0.0863 | Yes |
| 6 | HIST1H4C |  |  | 9 | 0.603 | 0.0996 | Yes |
| 7 | CENPU |  |  | 10 | 0.588 | 0.1146 | Yes |
| 8 | HIST1H1C |  |  | 11 | 0.583 | 0.1295 | Yes |
| 9 | HIST1H1D |  |  | 12 | 0.583 | 0.1444 | Yes |
| 10 | FAM111A |  |  | 13 | 0.576 | 0.1590 | Yes |
| 11 | EZH2 |  |  | 14 | 0.574 | 0.1737 | Yes |
| 12 | CLSPN |  |  | 17 | 0.561 | 0.1840 | Yes |
| 13 | C21orf58 |  |  | 18 | 0.559 | 0.1982 | Yes |
| 14 | RRM2 |  |  | 19 | 0.542 | 0.2120 | Yes |
| 15 | HMGB2 |  |  | 20 | 0.534 | 0.2257 | Yes |
| 16 | XRCC2 |  |  | 26 | 0.495 | 0.2282 | Yes |
| 17 | CHAF1A |  |  | 27 | 0.494 | 0.2408 | Yes |
| 18 | UBE2T |  |  | 31 | 0.475 | 0.2468 | Yes |
| 19 | GADD45G |  |  | 33 | 0.467 | 0.2567 | Yes |
| 20 | HIST1H1B |  |  | 35 | 0.460 | 0.2665 | Yes |
| 21 | GMNN |  |  | 38 | 0.453 | 0.2740 | Yes |
| 22 | ORC6 |  |  | 39 | 0.450 | 0.2854 | Yes |
| 23 | MMS22L |  |  | 40 | 0.448 | 0.2969 | Yes |
| 24 | ESCO2 |  |  | 43 | 0.440 | 0.3041 | Yes |
| 25 | UHRF1 |  |  | 45 | 0.436 | 0.3132 | Yes |
| 26 | RAD51AP1 |  |  | 46 | 0.435 | 0.3243 | Yes |
| 27 | PCNA |  |  | 48 | 0.435 | 0.3333 | Yes |
| 28 | TMPO |  |  | 49 | 0.432 | 0.3444 | Yes |
| 29 | DTL |  |  | 50 | 0.430 | 0.3553 | Yes |
| 30 | TOP2A |  |  | 52 | 0.426 | 0.3642 | Yes |
| 31 | SPC25 |  |  | 53 | 0.415 | 0.3747 | Yes |
| 32 | BRCA1 |  |  | 54 | 0.410 | 0.3852 | Yes |
| 33 | WDR76 |  |  | 56 | 0.403 | 0.3935 | Yes |
| 34 | TYMS |  |  | 60 | 0.390 | 0.3974 | Yes |
| 35 | PHIP |  |  | 61 | 0.387 | 0.4072 | Yes |
| 36 | RRM1 |  |  | 62 | 0.386 | 0.4171 | Yes |
| 37 | SMC2 |  |  | 63 | 0.386 | 0.4269 | Yes |
| 38 | E2F7 |  |  | 69 | 0.377 | 0.4265 | Yes |
| 39 | CSRP2 |  |  | 72 | 0.376 | 0.4320 | Yes |
| 40 | PSIP1 |  |  | 73 | 0.374 | 0.4416 | Yes |
| 41 | DEK |  |  | 74 | 0.373 | 0.4511 | Yes |
| 42 | SMC4 |  |  | 78 | 0.365 | 0.4543 | Yes |
| 43 | ITGB3BP |  |  | 79 | 0.364 | 0.4636 | Yes |
| 44 | CDCA5 |  |  | 81 | 0.361 | 0.4708 | Yes |
| 45 | STMN1 |  |  | 85 | 0.357 | 0.4739 | Yes |
| 46 | BARD1 |  |  | 86 | 0.357 | 0.4830 | Yes |
| 47 | MIS18BP1 |  |  | 89 | 0.355 | 0.4880 | Yes |
| 48 | USP1 |  |  | 92 | 0.352 | 0.4929 | Yes |
| 49 | ATAD5 |  |  | 97 | 0.345 | 0.4936 | Yes |
| 50 | NUSAP1 |  |  | 100 | 0.340 | 0.4983 | Yes |
| 51 | CKB |  |  | 102 | 0.335 | 0.5048 | Yes |
| 52 | NCAPD3 |  |  | 103 | 0.334 | 0.5133 | Yes |
| 53 | MCM8 |  |  | 104 | 0.331 | 0.5217 | Yes |
| 54 | ZNF43 |  |  | 107 | 0.330 | 0.5261 | Yes |
| 55 | INSM1 |  |  | 108 | 0.329 | 0.5345 | Yes |
| 56 | H2AFX |  |  | 115 | 0.322 | 0.5306 | Yes |
| 57 | HIRIP3 |  |  | 116 | 0.321 | 0.5388 | Yes |
| 58 | TIMELESS |  |  | 119 | 0.319 | 0.5429 | Yes |
| 59 | CCDC34 |  |  | 120 | 0.318 | 0.5510 | Yes |
| 60 | FRMD4B |  |  | 123 | 0.313 | 0.5550 | Yes |
| 61 | HMGN2 |  |  | 126 | 0.312 | 0.5589 | Yes |
| 62 | FBXO5 |  |  | 130 | 0.308 | 0.5607 | Yes |
| 63 | H3F3A |  |  | 132 | 0.304 | 0.5664 | Yes |
| 64 | KIF15 |  |  | 136 | 0.302 | 0.5681 | Yes |
| 65 | NCAPG |  |  | 137 | 0.301 | 0.5758 | Yes |
| 66 | RFC4 |  |  | 138 | 0.300 | 0.5834 | Yes |
| 67 | CENPK |  |  | 139 | 0.299 | 0.5910 | Yes |
| 68 | LMNB1 |  |  | 142 | 0.293 | 0.5945 | Yes |
| 69 | ASCL1 |  |  | 145 | 0.290 | 0.5978 | Yes |
| 70 | CCND2 |  |  | 149 | 0.288 | 0.5991 | Yes |
| 71 | NFIX |  |  | 152 | 0.286 | 0.6024 | Yes |
| 72 | RFC3 |  |  | 156 | 0.283 | 0.6036 | Yes |
| 73 | CEP78 |  |  | 159 | 0.281 | 0.6067 | Yes |
| 74 | ZNF93 |  |  | 160 | 0.281 | 0.6139 | Yes |
| 75 | CBFA2T2 |  |  | 162 | 0.280 | 0.6190 | Yes |
| 76 | H2AFY |  |  | 165 | 0.278 | 0.6221 | Yes |
| 77 | CHD7 |  |  | 166 | 0.277 | 0.6291 | Yes |
| 78 | VRK1 |  |  | 169 | 0.275 | 0.6321 | Yes |
| 79 | HELLS |  |  | 171 | 0.271 | 0.6370 | Yes |
| 80 | MAD2L1 |  |  | 174 | 0.270 | 0.6398 | Yes |
| 81 | NKAIN3 |  |  | 175 | 0.268 | 0.6467 | Yes |
| 82 | SNCAIP |  |  | 182 | 0.264 | 0.6413 | No |
| 83 | NCAPG2 |  |  | 187 | 0.260 | 0.6399 | No |
| 84 | CDK1 |  |  | 191 | 0.255 | 0.6403 | No |
| 85 | E2F3 |  |  | 194 | 0.252 | 0.6427 | No |
| 86 | CLIC1 |  |  | 237 | -0.261 | 0.5647 | No |
| 87 | SGTB |  |  | 239 | -0.261 | 0.5693 | No |
| 88 | FDFT1 |  |  | 243 | -0.262 | 0.5700 | No |
| 89 | ID4 |  |  | 261 | -0.267 | 0.5425 | No |
| 90 | PHLDA1 |  |  | 295 | -0.274 | 0.4830 | No |
| 91 | ABHD2 |  |  | 296 | -0.275 | 0.4900 | No |
| 92 | TMEM158 |  |  | 315 | -0.284 | 0.4609 | No |
| 93 | NUDT4 |  |  | 321 | -0.285 | 0.4581 | No |
| 94 | HMGCR |  |  | 418 | -0.334 | 0.2731 | No |
| 95 | LDLR |  |  | 419 | -0.334 | 0.2816 | No |
| 96 | MYC |  |  | 421 | -0.336 | 0.2882 | No |
| 97 | PTPRE |  |  | 469 | -0.372 | 0.2029 | No |
| 98 | LDHA |  |  | 498 | -0.412 | 0.1570 | No |
| 99 | FJX1 |  |  | 501 | -0.418 | 0.1636 | No |
| 100 | TGIF1 |  |  | 502 | -0.421 | 0.1743 | No |
| 101 | DANCR |  |  | 547 | -0.516 | 0.0988 | No |
Table: GSEA details [plain text format]

  

Fig 2: RICHARDS\_NOWAKOWSKI\_SCIENCE\_2017\_IPC\_DIV1\_UPREG: Random ES distribution      
 Gene set null distribution of ES for **RICHARDS\_NOWAKOWSKI\_SCIENCE\_2017\_IPC\_DIV1\_UPREG**
